# Supplementary material for: Defining the Relative Role of Insulin Clearance in Early Dysglycemia in Relation to Insulin Sensitivity and Insulin Secretion: The Microbiome and Insulin Longitudinal Evaluation Study (MILES)
Source: Metabolites. 2021 Jun 26;11(7):420. doi: 10.3390/metabo11070420 (PMC8304591; doi:10.3390/metabo11070420)
Supplement: Supplementary file 1 [file metabolites-11-00420-s001.zip › metabolites-1154026-SI.pdf]

Supplementary Table S1. Insulin homeostasis traits in African American individuals with normal glucose tolerance and dysglycemia.

	Normal glucose tolerance (n=65)	Dysglycemia (n=64)	P value
Insulin sensitivity	4.66 (3.65)	2.57 (1.82)	<0.0001
Insulin secretion	0.50 (0.44)	0.48 (0.38)	0.51
Disposition index	2.24 (1.06)	1.11 (0.75)	<0.0001
Insulin clearance	0.087 (0.041)	0.084 (0.041)	0.30

Supplementary Table S2. Insulin homeostasis traits in non-Hispanic White individuals with normal glucose tolerance and dysglycemia.

	Normal glucose tolerance (n=124)	IFG (n=100)	P value
Insulin sensitivity	6.15 (4.82)	3.03 (2.35)	<0.0001
Insulin secretion	0.31 (0.19)	0.33 (0.31)	0.25
Disposition index	1.84 (1.15)	0.97 (0.65)	<0.0001
Insulin clearance	0.13 (0.045)	0.11 (0.043)	<0.0001

Supplementary Table S3. Insulin homeostasis traits in individuals with normal glucose tolerance and prediabetes.

	Normal glucose tolerance (n=189)	PreDM (n=136)	P value
Insulin sensitivity	5.57 (4.59)	3.08 (2.32)	<0.0001
Insulin secretion	0.35 (0.29)	0.38 (0.39)	0.081
Disposition index	1.93 (1.10)	1.10 (0.62)	<0.0001
Insulin clearance	0.11 (0.054)	0.093 (0.045)	<0.0001

Supplementary Table S4. Insulin homeostasis traits in individuals with normal glucose tolerance and impaired glucose tolerance.

	Normal glucose tolerance (n=189)	IGT (n=63)	P value
Insulin sensitivity	5.57 (4.59)	2.67 (2.42)	<0.0001
Insulin secretion	0.35 (0.29)	0.33 (0.33)	0.74
Disposition index	1.93 (1.10)	0.92 (0.38)	<0.0001
Insulin clearance	0.11 (0.054)	0.096 (0.051)	0.0005

Supplementary Table S5. Insulin homeostasis traits in individuals with normal glucose tolerance and impaired fasting glucose.

	Normal glucose tolerance (n=189)	IFG (n=109)	P value
Insulin sensitivity	5.57 (4.59)	2.76 (2.31)	<0.0001
Insulin secretion	0.35 (0.29)	0.40 (0.38)	0.030
Disposition index	1.93 (1.10)	1.10 (0.71)	<0.0001
Insulin clearance	0.11 (0.054)	0.092 (0.042)	<0.0001

Supplementary Table S6. Parameter estimates and area under the receiver operating characteristics curve for logistic regression models examining the association of four insulin homeostasis traits with prediabetes, in separate models and for models combining traits.

	Base model	Model 1	Model 2	Model 3	Model 4	Model 5	Model 6
Age	0.039** (0.014)	0.014 (0.016)	0.039** (0.014)	0.035* (0.014)	0.002 (0.017)	-0.012 (0.018)	-0.007 (0.017)
Sex (male)	0.584* (0.241)	0.207 (0.278)	0.591* (0.243)	0.511* (0.246)	0.632* (0.289)	0.326 (0.307)	0.532 (0.299)
BMI	0.422** (0.130)	-0.338* (0.172)	0.432** (0.143)	0.275 (0.141)	0.121 (0.158)	-0.418* (0.198)	-0.156 (0.179)
Race (African American)	0.125 (0.257)	-0.140 (0.294)	0.135 (0.263)	-0.138 (0.278)	0.781* (0.325)	0.468 (0.361)	0.342 (0.350)
Insulin sensitivity		-1.568*** (0.218)				-3.278*** (0.408)	
Insulin secretion			-0.024 (0.138)			-1.824*** (0.309)	
Insulin clearance				-0.394** (0.146)		0.116 (0.298)	-0.690*** (0.184)
Disposition index					-1.951*** (0.240)		-2.112*** (0.259)
AUROC	0.654 (0.596-0.712)	0.799 (0.749-0.852)	0.655 (0.594-0.716)	0.676 (0.618-0.734)	0.846 (0.801-0.886)	0.871 (0.831-0.908)	0.860 (0.818-0.900)
AIC	426.245	356.295	428.214	420.708	319.964	299.530	306.596

*P<0.05; **P<0.01; ***P<0.001

Parameter estimates are listed with standard error in parentheses.

Supplementary Table S7. Comparisons of AUROC values from different logistic regression models for prediabetes.

	Base model	Model 1	Model 2	Model 3	Model 4	Model 5	Model 6
Base model	-	-4.92	-0.68	-1.08	-6.04	-6.85	-6.60
Model 1: base + insulin sensitivity	8.74×10^{-7}	-	4.81	5.603	-1.37	-3.73	-2.42
Model 2: base + insulin secretion	0.50	1.54×10^{-6}	-	-0.98	-6.07	-6.81	-6.60
Model 3: base + insulin clearance	0.28	2.10×10^{-8}	0.33	-	-5.19	-6.76	-6.41
Model 4: base + disposition index	1.59×10^{-9}	0.17	1.29×10^{-9}	2.06×10^{-7}	-	-1.90	-1.61
Model 5: base + insulin sensitivity, secretion, clearance	7.65×10^{-12}	1.89×10^{-4}	9.63×10^{-12}	1.34×10^{-11}	0.057	-	1.31
Model 6: base + insulin clearance, disposition index	4.11×10^{-11}	0.016	4.25×10^{-11}	1.43×10^{-10}	0.11	0.19	-

Values above the diagonal (upper right) are the Z scores. Values below the diagonal (lower left) are the P values.

Supplementary Table S8. Parameter estimates and area under the receiver operating characteristics curve for logistic regression models examining the association of four insulin homeostasis traits with impaired glucose tolerance, in separate models and for models combining traits.

	Base model	Model 1	Model 2	Model 3	Model 4	Model 5	Model 6
Age	0.092*** (0.020)	0.068** (0.023)	0.094*** (0.020)	0.090*** (0.020)	0.058* (0.028)	0.029 (0.030)	0.051 (0.030)
Sex (male)	0.125 (0.329)	-0.566 (0.409)	0.186 (0.333)	0.050 (0.336)	-0.126 (0.452)	-0.620 (0.502)	-0.215 (0.481)
BMI	0.540** (0.177)	-0.387 (0.238)	0.645*** (0.195)	0.375 (0.192)	0.058 (0.238)	-0.791* (0.344)	-0.382 (0.297)
Race (African American)	0.266 (0.341)	0.014 (0.402)	0.374 (0.352)	-0.003 (0.366)	1.263* (0.498)	0.878 (0.553)	0.881 (0.547)
Insulin sensitivity		-1.802*** (0.303)				-4.773*** (0.743)	
Insulin secretion			-0.249 (0.180)			-3.015*** (0.515)	
Insulin clearance				-0.412* (0.189)		-0.006 (0.395)	-0.799** (0.262)
Disposition index					-3.049*** (0.444)		-3.128*** (0.466)
AUROC	0.725 (0.654-0.792)	0.849 (0.793-0.907)	0.730 (0.656-0.801)	0.743 (0.674-0.810)	0.920 (0.879-0.958)	0.934 (0.894-0.970)	0.929 (0.886-0.968)
AIC	260.374	209.566	260.447	257.514	153.513	143.476	144.909

*P<0.05; **P<0.01; ***P<0.001

Parameter estimates are listed with standard error in parentheses.

Supplementary Table S9. Comparisons of AUROC values from different logistic regression models for impaired glucose tolerance.

	Base model	Model 1	Model 2	Model 3	Model 4	Model 5	Model 6
Base model	-	-3.74	-0.084	-1.00	-5.90	-6.04	-6.11
Model 1: base + insulin sensitivity	1.87×10^{-4}	-	3.271	3.90	-2.50	-4.02	-3.18
Model 2: base + insulin secretion	0.93	0.001	-	-0.62	-6.09	-5.88	-6.10
Model 3: base + insulin clearance	0.32	9.56×10^{-5}	0.536	-	-5.44	-5.93	-5.96
Model 4: base + disposition index	3.57×10^{-9}	0.012	1.17×10^{-9}	5.21×10^{-8}	-	-0.97	-1.47
Model 5: base + insulin sensitivity, secretion, clearance	1.58×10^{-9}	5.74×10^{-5}	4.20×10^{-9}	2.4×10^{-9}	0.033	-	0.36
Model 6: base + insulin clearance, disposition index	1.03×10^{-9}	0.001	1.10×10^{-9}	2.58×10^{-9}	0.14	0.72	-

Values above the diagonal (upper right) are the Z scores. Values below the diagonal (lower left) are the P values.

Supplementary Table S10. Parameter estimates and area under the receiver operating characteristics curve for logistic regression models examining the association of four insulin homeostasis traits with impaired fasting glucose, in separate models and for models combining traits.

	Base model	Model 1	Model 2	Model 3	Model 4	Model 5	Model 6
Age	0.031* (0.015)	-0.001 (0.017)	0.031* (0.015)	0.026 (0.015)	-0.008 (0.018)	-0.027 (0.019)	-0.022 (0.019)
Sex (male)	0.864*** (0.258)	0.532 (0.298)	0.866*** (0.261)	0.795** (0.263)	0.986** (0.311)	0.721* (0.330)	0.917** (0.324)
BMI	0.556*** (0.146)	-0.217 (0.189)	0.558*** (0.159)	0.406** (0.157)	0.304 (0.176)	-0.262 (0.213)	0.003 (0.195)
Race (African American)	0.050 (0.276)	-0.215 (0.320)	0.053 (0.283)	-0.228 (0.301)	0.682 (0.349)	0.401 (0.395)	0.228 (0.375)
Insulin sensitivity		-1.650*** (0.238)				-3.358*** (0.446)	
Insulin secretion			-0.005 (0.149)			-1.787*** (0.336)	
Insulin clearance				-0.410* (0.161)		0.152 (0.347)	-0.764*** (0.204)
Disposition index					-1.933** (0.255)		-2.218*** (0.279)
AUROC	0.689 (0.623-0.746)	0.820 (0.770-0.873)	0.689 (0.628-0.747)	0.707 (0.642-0.761)	0.854 (0.809-0.894)	0.880 (0.839-0.919)	0.872 (0.831-0.911)
AIC	369.753	304.727	371.752	365.034	278.681	257.084	265.323

*P<0.05; **P<0.01; ***P<0.001

Parameter estimates are listed with standard error in parentheses.

Supplementary Table S11. Comparisons of AUROC values from different logistic regression models for impaired fasting glucose.

	Base model	Model 1	Model 2	Model 3	Model 4	Model 5	Model 6
Base model	-	-4.53	0.31	-0.85	-5.41	-6.07	-5.93
Model 1: base + insulin sensitivity	5.84×10^{-06}	-	4.53	5.19	-1.06	-3.36	-2.17
Model 2: base + insulin secretion	0.76	5.99×10^{-6}	-	-0.85	-5.43	-6.07	-5.94
Model 3: base + insulin clearance	0.40	2.15×10^{-7}	0.39	-	-4.72	-6.06	-5.81
Model 4: base + disposition index	6.38×10^{-8}	0.29	5.77×10^{-8}	2.41×10^{06}	-	-1.89	-1.80
Model 5: base + insulin sensitivity, secretion, clearance	1.31×10^{-9}	7.71×10^{-4}	1.25×10^{-9}	1.39×10^{-9}	0.058	-	1.11
Model 6: base + insulin clearance, disposition index	3.05×10^{-9}	0.03	2.84×10^{-9}	6.40×10^{-9}	0.072	0.27	-

Values above the diagonal (upper right) are the Z scores. Values below the diagonal (lower left) are the P values.

Supplementary Table S12. Parameter estimates and area under the receiver operating characteristics curve for logistic regression models in non-Hispanic Whites examining the association of four insulin homeostasis traits with dysglycemia, in separate models and for models combining traits.

	Base model	Model 1	Model 2	Model 3	Model 4	Model 5	Model 6
Age	0.067*** (0.017)	0.031 (0.020)	0.067*** (0.017)	0.062*** (0.017)	0.008 (0.021)	-0.010 (0.023)	-0.001 (0.022)
Sex (male)	1.038*** (0.302)	0.704* (0.346)	1.062*** (0.305)	0.993** (0.310)	1.157** (0.374)	0.945* (0.395)	1.122** (0.387)
BMI	0.499*** (0.168)	-0.447 (0.240)	0.544** (0.185)	0.251 (0.190)	0.032 (0.217)	-0.640* (0.295)	-0.357 (0.262)
Insulin sensitivity		-1.662*** (0.285)				-3.418*** (0.599)	
Insulin secretion			-0.107 (0.175)			-2.005*** (0.401)	
Insulin clearance				-0.582** (0.200)		0.087 (0.416)	-0.770* (0.248)
Disposition index					-2.149*** (0.323)		-2.259*** (0.347)
AUROC	0.731 (0.667-0.797)	0.833 (0.78-0.889)	0.732 (0.669-0.796)	0.755 (0.688-0.818)	0.879 (0.831-0.918)	0.897 (0.851-0.934)	0.893 (0.845-0.929)
AIC	274.475	228.333	276.099	267.447	201.146	190.582	192.427

*P<0.05; **P<0.01; ***P<0.001

Parameter estimates are listed with standard error in parentheses.

Supplementary Table S13. Comparisons of AUROC values from different logistic regression models for dysglycemia in non-Hispanic Whites.

	Base model	Model 1	Model 2	Model 3	Model 4	Model 5	Model 6
Base model	-	-3.86	0	-1.31	-4.59	-5.16	-5.14
Model 1: base + insulin sensitivity	$1.12x10^{-4}$	-	3.65	4.21	-1.20	-2.97	-2.34
Model 2: base + insulin secretion	>.99	$2.60x10^{-4}$	-	-1.18	-4.729	-5.119	-5.16
Model 3: base + insulin clearance	0.19	$2.52x10^{-5}$	0.24	-	-3.77	-4.97	-4.90
Model 4: base + disposition index	$4.35x10^{-6}$	0.23	$2.26x10^{-6}$	$1.66x10^{-4}$	-	-1.48	-1.59
Model 5: base + insulin sensitivity, secretion, clearance	$2.42x10^{-7}$	0.002	$3.07x10^{-7}$	$6.78x10^{-7}$	0.14	-	0.58
Model 6: base + insulin clearance, disposition index	$2.77x10^{-7}$	0.019	$2.45x10^{-7}$	$9.5x10^{-7}$	0.11	0.56	-

Values above the diagonal (upper right) are the Z scores. Values below the diagonal (lower left) are the P values.

Supplementary Table S14. Parameter estimates and area under the receiver operating characteristics curve for logistic regression models in African Americans examining the association of four insulin homeostasis traits with dysglycemia, in separate models and for models combining traits.

	Base model	Model 1	Model 2	Model 3	Model 4	Model 5	Model 6
Age	0.005 (.021)	-0.002 (0.025)	0.005 (0.021)	0.005 (0.021)	-0.010 (0.028)	-0.023 (0.031)	-0.021 (0.030)
Sex (male)	-0.127 (0.383)	-0.512 (0.462)	-0.090 (0.386)	-0.155 (0.387)	-0.269 (0.496)	-0.596 (0.541)	-0.378 (0.520)
BMI	0.412* (0.198)	-0.149 (0.234)	0.469** (0.205)	0.384* (0.203)	0.122 (0.247)	-0.288 (0.285)	-0.089 (0.272)
Insulin sensitivity		-1.686*** (.339)				-3.649*** (0.661)	
Insulin secretion			-0.229 (0.195)			-2.053*** (0.510)	
Insulin clearance				-0.129 (0.207)		0.064 (0.451)	-0.683* (0.285)
Disposition index					-2.014*** (0.362)		-2.270*** (0.406)
AUROC	0.633 (0.541-0.733)	0.802 (0.723-0.871)	0.632 (0.536-0.722)	0.630 (0.538-0.727)	0.865 (0.793-0.921)	0.897 (0.834-0.943)	0.882 (0.819-0.929)
AIC	181.781	148.259	182.385	183.388	125.151	116.391	121.047

*P<0.05; **P<0.01; ***P<0.001

Parameter estimates are listed with standard error in parentheses.

Supplementary Table S15. Comparisons of AUROC values from different logistic regression models for dysglycemia in African Americans.

	Base model	Model 1	Model 2	Model 3	Model 4	Model 5	Model 6
Base model	-	-2.88	0.46	-0.047	-4.19	-4.79	-4.49
Model 1: base + insulin sensitivity	0.004	-	2.67	3.09	-1.46	-3.03	-2.01
Model 2: base + insulin secretion	0.64	0.008	-	-0.35	-5.03	-5.08	-5.03
Model 3: base + insulin clearance	0.96	0.002	0.72	-	-4.00	-4.76	-4.44
Model 4: base + disposition index	$2.82 x 10^{-5}$	0.15	$4.8 x 10^{-7}$	$6.44 x 10^{-5}$	-	-1.695	-1.15
Model 5: base + insulin sensitivity, secretion, clearance	$1.69 x 10^{-6}$	0.002	$3.70 x 10^{-7}$	$1.98 x 10^{-6}$	0.090	-	1.33
Model 6: base + insulin clearance, disposition index	$7.28 x 10^{-6}$	0.045	$5.01 x 10^{-7}$	$9.03 x 10^{-6}$	0.25	0.18	-

Values above the diagonal (upper right) are the Z scores. Values below the diagonal (lower left) are the P values.
